# A platform trial in practice: adding a new experimental research arm to the ongoing confirmatory FLAIR trial in chronic lymphocytic leukaemia

**DOI:** 10.1186/s13063-020-04971-2

**Published:** 2021-01-08

**Authors:** Dena R. Howard, Anna Hockaday, Julia M. Brown, Walter M. Gregory, Susan Todd, Tahla Munir, Jamie B. Oughton, Claire Dimbleby, Peter Hillmen

**Affiliations:** 1 Leeds Institute of Clinical Trials Research, University of Leeds, Leeds, UK; 2 Department of Mathematics and Statistics, University of Reading, Reading, UK; 3 St James’s Institute of Oncology, St James’s University Hospital, Leeds, UK; 4 Section of Experimental Haematology, Leeds Institute of Cancer and Pathology, University of Leeds, Leeds, UK

**Keywords:** Adding treatment arms, Platform trial, Flexible design, Chronic lymphocytic leukaemia, Complex innovative design, Randomised controlled trial, Confirmatory hypotheses, Statistical methodology, Trial management, Multi-arm clinical trial

## Abstract

**Background:**

The FLAIR trial in chronic lymphocytic leukaemia has a randomised, controlled, open-label, confirmatory, platform design. FLAIR was successfully amended to include an emerging promising experimental therapy to expedite its assessment, greatly reducing the time to reach the primary outcome compared to running a separate trial and without compromising the validity of the research or the ability to recruit to the trial and report the outcomes. The methodological and practical issues are presented, describing how they were addressed to ensure the amendment was a success.

**Methods:**

FLAIR was designed as a two-arm trial requiring 754 patients. In stage 2, two new arms were added: a new experimental arm and a second control arm to protect the trial in case of a change in practice. In stage 3, the original experimental arm was closed as its planned recruitment target was reached. In total, 1516 participants will be randomised to the trial.

**Results:**

The changes to the protocol and randomisation to add and stop arms were made seamlessly without pausing recruitment. The statistical considerations to ensure the results for the original and new hypotheses are unbiased were approved following peer review by oversight committees, Cancer Research UK, ethical and regulatory committees and pharmaceutical partners. These included the use of concurrent comparators in case of any stage effect, appropriate control of the type I error rate and consideration of analysis methods across trial stages. The operational aspects of successfully implementing the amendments are described, including gaining approvals and additional funding, data management requirements and implementation at centres.

**Conclusions:**

FLAIR is an exemplar of how an emerging experimental therapy can be assessed within an existing trial structure without compromising the conduct, reporting or validity of the trial. This strategy offered considerable resource savings and allowed the new experimental therapy to be assessed within a confirmatory trial in the UK years earlier than would have otherwise been possible. Despite the clear efficiencies, treatment arms are rarely added to ongoing trials in practice. This paper demonstrates how this strategy is acceptable, feasible and beneficial to patients and the wider research community.

**Trial registration:**

ISRCTN Registry ISRCTN01844152. Registered on August 08, 2014

## Background

### Aims

Front-Line therapy in CLL: Assessment of Ibrutinib-containing Regimes (FLAIR) is a phase III, open-label, randomised, controlled trial (RCT) in patients with previously untreated chronic lymphocytic leukaemia (CLL) sponsored and managed by the University of Leeds. The primary aim of the trial when it was originally designed was to assess current standard therapy with fludarabine, cyclophosphamide and rituximab (FCR) against ibrutinib with rituximab (IR) in terms of progression-free survival (PFS). At the outset of the trial, 754 patients were planned to be randomised in 4 years, with primary outcomes being available after a further 4 years of follow-up. The protocol describing the trial as originally designed has been previously published [1]. Although the trial was designed to assess a single experimental arm, it was known that promising therapies were being assessed in early phase trials, and so the trial design was kept simple to more easily enable new experimental treatments to be added if appropriate. The trial opened to recruitment in September 2014, with 70 UK centres planned, and has recruited consistently ahead of target. During recruitment, early evidence emerged of another very promising treatment combination in this population, ibrutinib with venetoclax (I+V). In order to be able to assess I+V in a phase III trial in the same population in the UK in a timely manner, it was added into the existing FLAIR trial framework after 2 years and 10 months of the planned recruitment period, when target recruitment was at 84%.

This publication describes the methodological and practical issues involved in successfully amending the FLAIR trial to include this promising experimental therapy so that its assessment could be expedited into a phase III setting. We detail the pathway for amending this traditional RCT in order that it became a platform trial with a complex innovative design (CID), where the objective of a ‘platform trial’ is ‘to study multiple targeted therapies in the context of a single disease in a perpetual manner with therapies allowed to enter or leave the platform on the basis of a decision algorithm’ [2–5]. A platform trial generally falls under the umbrella topic of an ‘adaptive design’, which is described as a ‘clinical study design that uses accumulating data to decide how to modify aspects of the study as it continues, without undermining the validity and integrity of the trial’ [6]. However, we acknowledge that there are no completely accepted definitions of platform or adaptive trials. Furthermore, our design does not, strictly speaking, meet the definition of an adaptive design because the amendment is not informed by accumulating internal trial data. The benefits of this aspect are discussed in the ‘Analysis methods following adaptation of design features with combination of information across trial stages’ section.

It is described how the strategy used improved the efficiency and relevance of this confirmatory trial, reducing the time taken to answer new and important clinical questions without compromising the original design and maintaining statistical validity. Consequently, this type of amendment is not only acceptable to, but actively benefits patients, researchers, funders, regulators and the wider research community.

### The original FLAIR trial design

The original design of FLAIR was a phase III, multi-centre, randomised, controlled, open-label, parallel-group trial comparing IR against the current standard FCR, with a total of 754 participants to be randomised on a 1:1 basis over a 4-year recruitment period. FCR is given for a maximum of 6 cycles with each cycle being repeated every 28 days. Participants randomised to receive IR receive rituximab in the same schedule as for FCR, and ibrutinib daily for 6 years, until minimal residual disease (MRD) negativity stopping rules are reached or until disease progression or withdrawal.

**Fig. 1:**
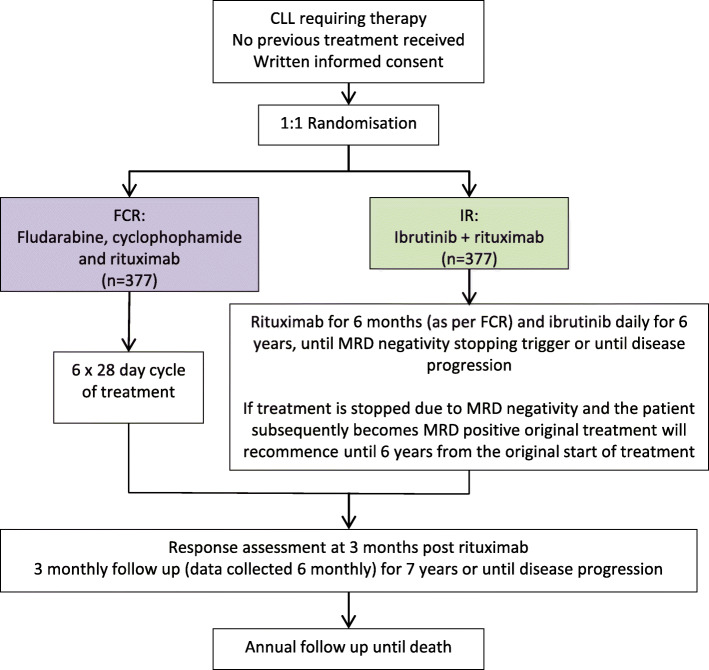
Participant pathway into FLAIR prior to the amendment. The experimental arm (IR) is shown in green and the control arm (FCR) in purple

The trial aims were primarily to provide evidence to assess whether IR is superior to FCR in terms of PFS and whether IR toxicity rates are favourable as a secondary endpoint. Other key secondary endpoints to be assessed included overall survival (OS), attainment of undetectable MRD, response to therapy, health-related quality of life (QoL) and cost-effectiveness, as well as an evaluation of discontinuation and re-start of ibrutinib therapy if indicated based on the levels of residual disease. Randomisation used minimisation with a random element to ensure treatment arms were well-balanced for the following participant characteristics: Binet stage (A progressive or B, C), age group (≤ 65 years, > 65 years), gender (male, female) and centre (all participating centres). Figure 1 illustrates the original participant pathway.

The sample size was based on testing the null hypothesis of no difference in PFS between the treatment arms. To test a superiority hazard ratio of 0.75 (from 4.5 [7] to 6 years), with an overall two-sided 5% significance level and 80% power, assuming 4 years recruitment and 4 years follow-up, allowing for 5% drop-out, and inflating for a planned formal interim analysis on PFS when half the numbers of events were observed, 754 participants were required to observe 379 events.

### Minimal residual disease negativity treatment break

MRD negativity is defined as the presence of < 0.01% CLL cells in the peripheral blood or bone marrow. The detection of MRD above this level after therapy is an independent predictor of outcome [8], where detectable disease is prognostic of progression. It is hypothesised that once a patient’s disease falls below a certain level, it may reach a point at which the CLL cells cannot grow back to being detectable or progressing to a level which requires therapy. In these patients, continuing treatment may be unnecessary. The FLAIR trial therefore includes an MRD negativity stopping rule, in which participants receiving ibrutinib who become MRD-negative stop therapy after a certain period of time, determined by the time it took to reach MRD negativity. If participants stop treatment due to MRD negativity and then relapse at the MRD level before the end of the trial treatment period, treatment is restarted to assess whether MRD eradication is re-achieved and to protect the primary endpoint of PFS. This is not considered a progression event.

### Funding and approvals

FLAIR is partially funded by Cancer Research UK following review and approval by their Clinical Trials Advisory and Award Committee (CTAAC) in November 2012. Janssen Pharmaceuticals provide ibrutinib free of charge for use in the trial and provide funding via an educational grant. The trial received ethical approval from the National Research Ethics Service (NRES) Committee Yorkshire and The Humber and regulatory approval from the Medicines and Healthcare Products Regulatory Authority (MHRA) in June 2014. The trial was registered on the ISRCTN registry (ISRCTN01844152) ahead of the first participant being recruited. An independent Data Monitoring and Ethics Committee (DMEC) and Trial Steering Committee (TSC) were established during the trial set-up and approved the original protocol and trial design. The DMEC and TSC both meet at least annually, and the DMEC review safety reports on a 3-monthly basis.

### Accrual

As with all clinical trials, recruitment is monitored closely by the Trial Management Group (TMG). The TMG decided that it would only be appropriate to add arms during recruitment if the trial recruited at least as well as anticipated and the addition of arms would not significantly delay the reporting timelines of the original design. By the end of 2015, it was clear that recruitment was going to continue at a rate that was 15–20% ahead of target, and the TMG agreed that this was sufficient for an amendment to add new arms.

### Considerations when designing the original FLAIR trial to enable amendments

Due to treatment advances, PFS times are increasing, and whilst clearly beneficial to patients, this presents challenges for research in ensuring that trials are feasible and the outcomes remain relevant in the face of a long-term endpoint. In addition, the drug development environment in CLL is rapidly changing. At the time of designing the original FLAIR trial, there was a series of phase II trials planned as part of the Bloodwise Trials Acceleration Programme (TAP) [9] run through the University of Birmingham assessing new treatment combinations with targeted therapies, some of which included ibrutinib. The new combinations were hypothesised to give deeper responses than IR, but there was very little evidence of activity or safety in patients with CLL. Our options at the time were to either wait for the phase II outcomes in case they were positive, delaying the phase III assessment of IR; start the trial as planned, which would saturate the UK population for the coming years and deny the investigation of a new promising combination in a phase III trial; or start the trial but plan to be able to amend it to include new treatment arms if appropriate once early phase data were available. It was clear that in order to speed up the investigation of promising new therapies and improve the efficiency of the phase III trials process in CLL to mirror that in phase II, the last option was necessary. For this reason, the FLAIR trial had a simple design so it could be more readily amended.

New treatment arms have rarely been added to ongoing confirmatory trials in practice [10], although phase III trials are the longest and most expensive part of the drug development process and doing so would greatly improve efficiency. The STAMPEDE trial paved the way in this type of design amendment [11–14], and here we describe our experiences in an alternative trials setting. The methodological and practical issues when incorporating a new experimental research arm into the FLAIR trial during recruitment are presented, describing how they were addressed to overcome barriers and ensure the amendment was a success.

## Methods: design of the FLAIR amendment

### The emerging combination: ibrutinib + venetoclax

By the end of 2015, early-stage data showed impressive response rates for venetoclax (V) (ABT-199) when given in combination with rituximab (V+R) in patients with relapsed/refractory CLL, and eradication of detectable MRD in 53% of patients, which had not previously been seen with any other targeted treatments [15]. Based on pre-clinical data, it was anticipated that the combination of V plus ibrutinib (I+V) would be highly synergistic given the complimentary modes of action of the two agents, as the ibrutinib arrests CLL cell proliferation and venetoclax is pro-apoptotic leading to their early cell death [16, 17]. Since FLAIR incorporates MRD negativity stopping rules designed to reduce long-term toxicities and treatment costs, it was important to identify a treatment combination with the greatest chance of inducing MRD negativity. It was hypothesised that the addition of venetoclax to ibrutinib would reduce MRD levels faster and more effectively than those expected with single-agent I or IR and therefore allow the duration of therapy based on the level of disease to be reduced, leading to a reduction in long-term resistance and toxicities and an overall cost saving. I+V was therefore chosen to be assessed in the phase II TAP trial ‘CLARITY’ (ISRCTN: 13751862) in 50 patients with relapsed CLL in a non-randomised setting. Preliminary results from CLARITY were expected to be available during the first half of 2017, by which time FLAIR would have recruited approximately two thirds of the planned sample size. The TMG agreed that these timelines were feasible to allow I+V to be added to FLAIR as a new arm but only if work began on designing the amendment and applying for approvals prior to the availability of the phase II safety or preliminary efficacy results from CLARITY. The approval applications were made with the caveat that they would be withdrawn if emerging data indicated. Preliminary safety and early efficacy data from the CLARITY trial became available in early 2017 and supported the investigation of I+V in a confirmatory setting [18].

### Inclusion of an ibrutinib monotherapy control arm

In order to protect the trial from changes in practice in the future, a single-agent ibrutinib arm (I) was also added alongside I+V as an additional control therapy. At the time of designing the amendment, FCR was still the standard of care in front-line CLL and thus was the required comparator for all experimental therapies. However, in 2016, the ibrutinib licence was extended to include use as a single agent for patients with previously untreated CLL. The TMG therefore felt that an ibrutinib-containing therapy could become standard of care in the FLAIR population before the trial was fully reported. As IR was hypothesised to reach deeper responses than single-agent ibrutinib, and was being assessed in clinical trials other than FLAIR, it was unclear whether IR or I alone was more likely to become the standard of care long term in the UK. It was therefore proposed to include a single-agent I comparator arm in addition to IR and FCR at the time of the amendment to add I+V. This mitigated the risk that the outcomes of the trial would be hugely devalued if it were to show that I+V was better than FCR, but FCR was no longer the standard of care.

In order to ensure the timely reporting of trial outcomes, it was not feasible to include both the IR and I alone arms as primary comparators to I+V in addition to FCR. In discussion with Cancer Research UK (CRUK), it was agreed that a decision would be taken at the end of the planned recruitment period to the IR vs FCR comparison to drop either IR or single-agent I. The decision on which arm to choose would primarily be made based on anticipated emerging MRD data from other trials that were due to report ahead of the decision point.

The amendment therefore included the addition of two new arms, one experimental and one control. In addition, there were two new primary hypotheses, one comparing I+V to FCR and the other comparing I+V to I or IR.

### Stage 2 amended trial design

The amended FLAIR design was a phase III, multi-centre, multi-arm, randomised, controlled, open, parallel group trial with participants randomised to receive FCR, IR, single-agent I or I+V on a 1:1:1:1 basis. The eligibility criteria remained unchanged from the original design as did the treatment schedules for FCR and IR. Participants randomised to I+V receive ibrutinib for 8 weeks before venetoclax is added over a 5-week dose-escalation phase. In the single-agent I, IR and I+V arms, ibrutinib and venetoclax (as relevant) are administered for 6 years, until the MRD negativity stopping rules are triggered or until disease progression or withdrawal. If treatment is stopped and restarted due to MRD levels, participants randomised to single-agent I or IR receive further single-agent I, and participants randomised to I+V receive further I+V. Abbvie provide venetoclax free of charge for use in the trial and an educational grant for the additional running costs associated with the new arms.

**Fig. 2:**
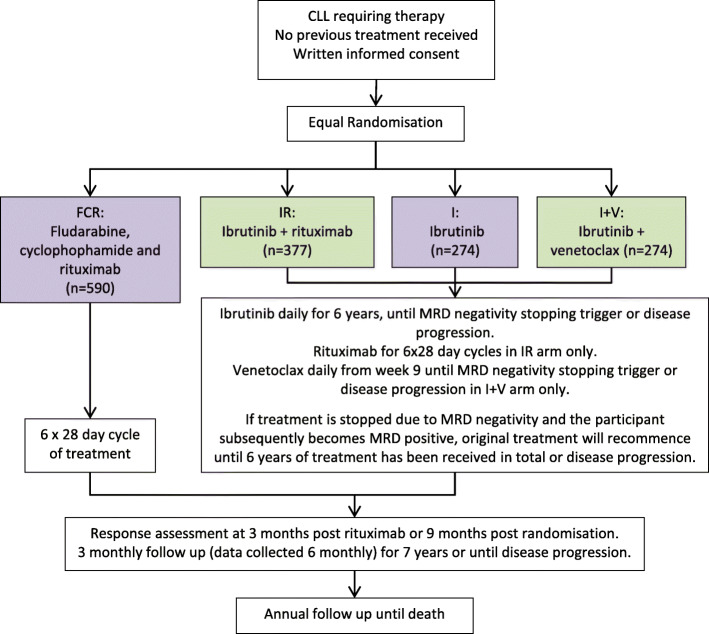
Participant pathway into the FLAIR trial during stage 2. The experimental arms (IR and I+V) are shown in green and the control arms (FCR and I) in purple. The primary objectives are: •To assess IR vs FCR in terms of PFS •To assess I+V vs FCR in terms of PFS •To assess I+V vs I or IR, as appropriate, in terms of MRD negativity rate at 24 months post-randomisation

The amended trial aims to provide evidence for the future first-line treatment of CLL patients by assessing whether IR is superior to FCR in terms of PFS, whether I+V is superior to FCR in terms of PFS, whether I+V is superior to I or IR (as appropriate) in terms of MRD negativity, and whether IR and I+V toxicity rates are favourable. The other key endpoints to be assessed remain unchanged from the previous design, but now also compare I+V with FCR and with I or IR. Figure 2 illustrates the amended participant pathway.

The sample size for I+V vs FCR was based on testing the primary null hypothesis of no difference in PFS between the treatment arms. The assumptions for the clinically relevant effect size differed from those for IR vs FCR due to the evidence published in 2015 comparing I with chlorambucil [19], demonstrating that ibrutinib monotherapy leads to a better PFS than thought in this population at the time of the original design. To assess a superiority hazard ratio of 0.69 (for a median PFS increase of 4.5 to 6.5 years) with an overall 5% significance and 80% power, assuming a 2.5-year recruitment period and 3.5 years of follow-up, and allowing for a 5% dropout rate, 274 participants were required to be concurrently randomised to each of FCR and I+V in order to observe 232 events. A total of 822 participants were therefore required to be concurrently randomised to FCR, I or IR, and I+V. A formal interim analysis on PFS was planned when half of the numbers of events (a total of 116 progressions and/or deaths) were observed in FCR and I+V, in order to allow large differences between the treatment arms to be reported early to the DMEC. The O’Brien and Fleming alpha-spending function [20] was used to account for testing at multiple time points to conserve the overall type I error.

This number of patients provides adequate power to compare I+V against I or IR in terms of the primary outcome MRD negativity rate. PFS is included as a key secondary endpoint, but it is confounded by the MRD stopping rule potentially affecting the duration of therapy differently in each arm. The analysis of MRD negativity will be carried out 2 years after the close of the recruitment. At the time of designing the amendment, the MRD negativity rates in the ibrutinib-containing arms were not known, so a range of power calculations were explored. With 260 evaluable patients in each of the arms and a 5% two-sided significance level, there is 90% power to detect an improvement from, say 10 to 20%. If there is a larger proportion that becomes MRD negative with IR, say 20%, there is 90% power to detect an improvement to 32.5%, and since a large increase in MRD negativity would be required in order to justify the addition of V, the planned number of patients is more than adequate.

### Dropping I or IR in stage 3

The decision on whether the most appropriate comparator for I+V would be I or IR, in addition to FCR, was discussed with the DMEC and TSC during February 2018, in order to make the amendment in July 2018 once recruitment to the original trial arms had completed. The emerging evidence from external trials suggested that IR was no better than I in terms of PFS [21] and also that IR did not lead to good enough rates of MRD negativity. In addition, MRD negativity results from IR participants in stage 1 of FLAIR were summarised for the DMEC, and these strengthened the external evidence. The DMEC were able to request this internal data to aid their decision because it was a non-comparative summary of a subset of a single arm and only involved participants who were external to the concurrent stage 2/3 population for the comparison under consideration. The DMEC had not reviewed any comparative efficacy data at the point of discussing the amended design. It was therefore agreed that the IR arm would be stopped, and the trial would continue to randomise on a 1:1:1 basis to FCR, I monotherapy and I+V. There were no changes to the existing treatment schedules or eligibility criteria.

### Overview of the trial stages

**Fig. 3:**
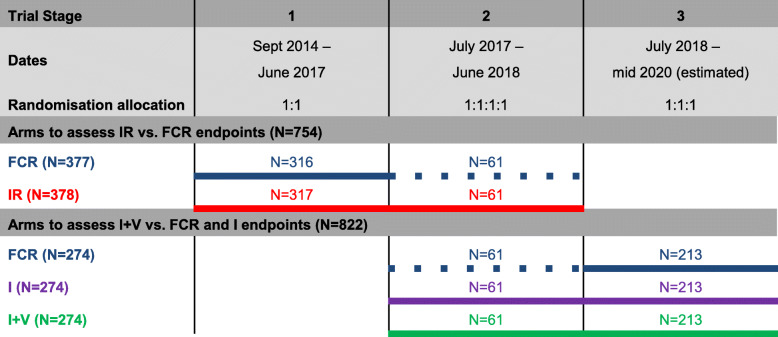
Overview of the trial stages

In total, 1516 participants will be randomised to the trial. A total of 754 participants are required to be randomised concurrently to FCR and IR (stages 1 and 2) and 822 participants to FCR, I and I+V (stages 2 and 3). In addition, 61 FCR patients in stage 2 are included in both randomisations, and therefore, the total sample size is less than it would have been in independent trials. Note that if it had instead been decided that the most appropriate comparator for I+V was IR, 61 patients would have been recruited to the single-agent I arm who would not be included in any primary analysis population; however, this was felt to be an acceptable risk to the overall strategy. Figure 3 outlines the treatment arms that are included over each stage in the trial. A dotted line indicates that the participants recruited to those arms are included in both sets of randomised comparisons.

The original FLAIR trial was planned to recruit in 4 years. Even with the addition of the extra arms, recruitment completed 2 months ahead of schedule. The amendment included additional funding to open more centres, so over 100 were opened rather than the 70 originally planned. In this way, the delivery of the original trial was not compromised by the amendment.

## Results

### Statistical considerations to ensure unbiased results

#### Concurrent comparisons

For the analysis of the trial, all primary and secondary endpoint comparisons will only include patients randomised contemporaneously. That is because if there is a shift in the patient population due to the design change or changes in practice over time, it may shift the median survival and could bias the results [22, 23]. Sixty-one FCR patients who were included in the original FLAIR design can also be used as comparators for the I+V arm, therefore reducing the numbers needed compared to a new trial. Data from non-concurrent patients across the whole protocol will be used to carry out exploratory investigations. There will be more similarities between these patients than those from separate trials, and having such a wealth of data on this population could allow subgroups of patients, for example those with certain genetic markers, to be investigated to generate hypotheses that could inform future research. This trial was not designed to report comparisons between the non-concurrent trial arms single-agent I vs IR, or I+V vs IR, once it had been decided that IR would be dropped from stage 3.

#### Type I error control

There are a number of ways the type I error could be inflated or bias introduced in a multi-arm platform trial design as follows.

##### Multiple primary hypotheses assessing I+V

The I+V arm is being assessed in two primary hypotheses: against FCR for PFS and against I for MRD negativity. In order for I+V to be deemed a ‘success’, it needs to be significantly better than both of its control groups. Where both hypotheses are required to be superior, there is no inflation of the type I error rate, and therefore, no adjustment is required [24].

##### Multiple hypothesis testing in the same protocol

This protocol allows the opportunity for both IR and I+V to be declared superior to the current standard within a primary analysis, therefore increasing the chance of a type I error for an ibrutinib-containing combination. Whilst both give the opportunity for a therapy containing ibrutinib to be declared superior, the aim of giving the additional treatments in combination with ibrutinib (rituximab and venetoclax) is to be able to take a break from ibrutinib therapy and in fact reduce the burden of ibrutinib compared to the likely future standard of care of continuous single-agent ibrutinib. Since a type I error for these comparisons does not directly benefit the same claim of effectiveness for an experimental therapy, family-wise type I error rate (FWER) control is not necessary for this reason [25]. If the two primary hypotheses had been assessed in separate protocols no adjustment would be required, and in this case, it is feasible to assume that the questions would have otherwise been assessed in different trials. Since there is an overlap in recruitment, some of the control data is shared between the IR and I+V vs FCR hypotheses. Howard et al. [25] show that the resulting correlation between the hypotheses reduces the overall type I error over what it would have been if they had been assessed independently, and therefore, FWER adjustment is also not necessary due to the sharing control data. In summary, adjusting for multiple testing due to assessing multiple experimental arms would be an ‘unnecessary penalty for efficiency’ [26]. This decision was approved by the DMEC, TSC and CRUK.

##### Multiple analysis time points

In order to account for the formal interim analyses allowing for early rejection of the null hypothesis for IR or I+V compared to FCR based on early evidence of efficacy, the O’Brien and Fleming alpha-spending function [20] was used. The method recommends that given a single interim analysis, the interim results are compared to a *p* value of 0.005, and the final results are then compared to a *p* value of 0.048. This is applied to each of the hypotheses separately.

##### Analysis methods following adaptation of design features with combination of information across trial stages

It can be seen from Fig. 3 that the trial consists of three stages, each with different randomisation options. The decision to add the new arms was made without reference to internal trial data. At the end of stage 2, after the IR vs FCR randomisation had reached its target recruitment, IR was dropped from the trial. The decision to drop IR rather than I was made primarily based on the data external to the trial, but also on a summary of MRD results from participants randomised to IR in stage 1 only. This has no implications for the analysis of the IR vs FCR comparison across stages 1 and 2 because the planned recruitment had completed at the time that the amendment to drop IR was made. It also has no implications for the analysis of the I+V vs I comparison across stages 2 and 3 because the data summarised was for the IR arm from stage 1 participants only, and these are external to the concurrent randomisation across stages 2 and 3. In addition, summarising a subset of MRD results for IR patients for the DMEC does not affect the type I error for the final analysis of PFS for IR vs FCR because no randomised comparison was carried out.

Since the decision to add (and stop) arms was not informed by any analysis of data internal to the existing hypotheses at the time of the amendment, the trial is not truly adaptive because the second stage is not informed by the first stage data for each hypothesis. Therefore, adaptive analysis methods are not required, and each hypothesis is analysed by pooling the data over the relevant stages. A multivariable Cox regression is planned to analyse the PFS primary endpoints, and a multivariable logistic regression is planned for the binary primary endpoint of achievement of MRD negativity. Whilst the key eligibility criteria did not vary across the stages, it is possible that the different treatment options attract slightly different patients. In the first stage, there was a 50% chance of receiving ibrutinib (IR); in the second stage, this increased to a 75% chance (IR, I or I+V); and in the third stage, 67% (I or I+V). In addition, the number of centres increased leading up to the second stage. In case of any stage effects caused by the changing treatment options or centres, the planned multivariable regressions for all analyses of primacy include ‘trial stage’ as a covariate as well as the stratification factors: disease stage, age group and gender [27].

#### Other statistical details

In addition to the key points detailed above, the following were also considered to ensure the ability of the trial to answer all the primary hypotheses.

##### Power

The new hypotheses comparing I+V concurrently against FCR and I were both adequately powered. The design for the original hypothesis was unchanged by the addition of the new arms, so the power calculation remained appropriate. In addition, since there was no adjustment to the significance level for multiple hypothesis testing, the power remained adequate.

##### Randomisation and allocation

Randomisation was by minimisation with stratification and a random element [28], and the stratification factors were unaltered for the duration of the trial. At each stage, the minimisation algorithm was reset. This was felt appropriate so that each set of concurrent data within each stage remains balanced, and there were enough patients in each stage that the arms were generally well balanced overall. Continuing the minimisation algorithm would not be appropriate when adding an arm as all totals would be zero for the new arm at first, distorting the algorithm. There is no reason why resetting the minimisation algorithm would introduce any bias.

It was decided to maintain an even allocation ratio to all arms in all stages, regardless of the number of experimental treatments. There are views in the literature [10, 29] that randomising a higher proportion to control can be more efficient in terms of total patient numbers needed when there is more than one experimental arm, although this is less clear when there are multiple control arms, or that all arms should complete recruitment at the same time to avoid a third stage. However, having a different allocation ratio in different stages for a single hypothesis would complicate the power calculation and affect the analysis [22].

### Implementation and operational requirements

#### Timelines

The timeline of key events from opening the FLAIR trial and deciding to progress the amendment to opening the new arms are summarised in Table 1. Almost as soon as stage 1 opened to recruitment, discussions began about a possible amendment. The decision to progress the amendment to include an I+V experimental arm was taken after the trial had been open for 1 year. It took a further 2 years before stage 2 opened. These timelines were driven by the lack of availability of the early-phase data until mid-2017, but it would have been difficult to reduce this set-up time much for reasons described below.

**Table 1:** Timeline of events from trial opening to adding the new arms

Date	Task
Q3 2014	**Stage 1 open to recruitment September 2014: FCR vs IR**
Q3 2015	Emerging response data for venetoclax FLAIR recruiting ahead of target Decision to progress amendment
Q4 2015	Initial DMEC/TSC review and provisional approval CRUK amendment application AbbVie funding application NCRI CSG review
Q1 2016	AbbVie agreed to support amendment subject to contract
Q2 2016	CRUK approval Janssen agreed to support amendment subject to contract Protocol amendments began
Q1 2017	Amendments to protocol and PIS finalised DMEC/TSC final approval MHRA substantial amendments submitted
Q2 2017	Ethics substantial amendments submitted Preliminary safety data for I+V became available CRFs and database amendments finalised Contracts signed and all approvals had been received
Q3 2017	**Stage 2 open to recruitment: FCR vs IR vs I vs I+V** Amendment randomisation became live July 2017 Original randomisation system switched off August 2017

#### Approvals and funding

##### Oversight committees

The concept of adding new arms to FLAIR was initially discussed with the DMEC and TSC in October 2015. Neither committee had seen any internal efficacy data; the DMEC had only reviewed safety data, and the formal interim efficacy analysis was not triggered until after the implementation of both amendments. Both groups gave their approval for the necessary funding applications to proceed and agreed that they would approve the new design once funding was in place. The TSC includes a patient and public involvement (PPI) representative who was actively involved in the discussions and the decision to approve the amendment and felt that the efficiency of the design would be beneficial to patients. The final amended trial design was reviewed and approved by the oversight committees in February 2017.

The amendment was also presented to and approved by the NCRI CLL Subgroup Committee and the NCRI Haemato-oncology Clinical Studies Group (CSG). Both groups were very supportive of the amended design, which was important as they represent the FLAIR principal investigators from participating centres and patient groups.

##### Cancer Research UK

A no-cost amendment application was submitted to the CRUK Clinical Research Committee in November 2015 for review by the committee in May 2016. This process included an international peer review by four reviewers. One of the peer reviewers identified I+V as a combination with ‘game-changing potential’ and another that ‘with the amended design of adding ibrutinib and ibrutinib + venetoclax arms, this trial has the potential to help define the standard for frontline CLL treatment worldwide’. Some of the reviewers supported the design methodology of adding new arms with one saying ‘As the availability for novel agents increases across all types of cancer, studies such as this can be looked at as a model for efficiently answering key questions in a field.’ and another that ‘The planned amendment is essential for this trial to ensure that the conclusions remain relevant when it is due to report’. However, others were concerned about the complexity of the amended trial design and if this would impact deliverability, whether it was reasonable not to adjust for multiple testing given the shared control patients, whether the design would be supported by the relevant pharmaceutical companies and whether changes in practice could affect the trial long term. All of these points were addressed to the satisfaction of the committee, and approval was granted.

##### Pharmaceutical companies

Due to the relatively short timelines between I+V emerging as an important treatment combination and the original FLAIR design meeting the recruitment target, discussions with pharmaceutical companies had to happen in parallel with the amendment application to CRUK. The amended design included the use of the new Investigational Medicinal Product (IMP), venetoclax, manufactured by Abbvie and a considerably higher number of patients receiving ibrutinib. In advance of the design being discussed with the DMEC, TSC and NCRI committees, initial discussions had been held with Abbvie to establish provisional support for the design. A formal funding application was submitted to them in November 2015, and in February 2016, Abbvie agreed to provide free venetoclax and an educational grant for the additional running costs associated with the new arms, subject to successful contract negotiation. In June 2016, Janssen, the manufacturer of ibrutinib, agreed to provide free ibrutinib for the additional participants in the new arms and associated IMP distribution costs.

To finalise this additional support, a contract amendment was required with Janssen and a new contract was required for Abbvie. These contracts were both signed in May 2017. Contract negotiation is a common factor impacting trial set-up times and delaying trials opening to recruitment. These negotiations are made more complex by having multiple pharmaceutical funders and negotiating contracts that comply with charitable funders’ terms and conditions. It was arguably simpler adding an additional pharmaceutical partner after the trial had opened as the principles around data sharing and intellectual property had already been agreed with one company, so there was an understanding that those terms would be equivalent for new funders.

##### Ethical and regulatory

Protocol development, including associated documentation such as the participant information sheet (PIS), was finalised in February 2017 following a number of reviews both by the TMG and by pharmaceutical companies. The PIS was also reviewed by the PPI representatives on the NCRI CLL Subgroup Committee. Substantial amendments were submitted to the MHRA and ethics committee in March and April 2017, respectively. Ethics approval was received promptly within 2 weeks of the submission, but MHRA approval was not received until May 2017. This was delayed as the MHRA requested additional information about the safety of the I+V combination.

#### Data management considerations

The trial case record forms (CRFs) were updated in line with the trial protocol and were finalised in April 2017. It was decided to amend the existing trial database rather than having a separate database for the new comparisons. This added some limitations in terms of how data for the new arms were collected as it needed to work within the existing database structure, but did not compromise the quality of the data that was collected for the analyses in terms of data compliance or the number of data queries raised. A new randomisation system was implemented for the four-arm design which meant all centres had to be re-activated on the system.

#### Implementation at centres

A key consideration when the amendment was designed was that the addition of new arms should not significantly delay the reporting of the FCR vs IR comparison beyond the original planned timelines. As the trial was recruiting ahead of target, and the number of recruiting centres was planned to be increased from 70 to 110, the impact on the original analysis timelines was minimal. Set-up of the additional centres started ahead of the amendment opening to further increase the recruitment rate. Five existing centres decided not to participate in the amended trial, four due to the lack of capacity and one because they were unable to cover the cost of MRD testing which was allocated as a treatment cost.

The new randomisation system went live at the beginning of July 2017. Thirty-nine centres opened to the new design within the first week. It was agreed that the old randomisation system would be switched off at the end of August 2017 with all centres needing to have approvals for the amendment in place before then or they would have been suspended to recruitment. Sixty-eight centres opened before the original randomisation system was closed, rising to over 100 centres in the following months.

## Discussion

The strategy of incorporating a new experimental treatment into the FLAIR framework was successful and hugely advantageous, without compromising either the original or new research goals. In this way, we were able to incorporate emerging evidence to test two experimental therapies instead of one, keeping the trial outputs timely and relevant and minimising resources. There are many advantages to this strategy, and although there were also challenges, these were not insurmountable.

### Challenges

#### Perceived risk

Adapting a trial in any way introduces complexities, both real and perceived. A comment from a CRUK peer reviewer was ‘Trial design is now more complex, so additional risk that not all components will be completed as planned’. This general feeling that the more complex the design, the more risk is involved both operationally and statistically was echoed in the discussions with clinical and patient representative members of the NCRI CLL Subgroup. Whilst a larger and longer trial with more components will naturally carry more risk, the trials team were careful to consider any potential sources of bias or disadvantages and address them, as discussed throughout this manuscript. The original trial question of IR vs FCR is largely unaffected by the addition of the new arms. The number of planned centres were increased from 70 to 110 to ensure that recruitment to the original arms was not negatively impacted by the addition of the new arms, and in fact, this comparison recruited ahead of target even with the amendment. The analysis is planned when the data in these arms are mature and without reference to the new arms, so the trial outcomes are not delayed by the amendment. The analysis includes trial stage as a covariate to account for any potential changes to the population caused by adding the arms and centres. Each primary hypothesis is fully powered and is assessed based on concurrently recruited patients only, which protects against changes in the trial population over time, and statistical aspects relating to error rates due to sharing a protocol and control data have also been considered in detail as described previously.

### Timelines of implementation

In order for the confirmatory assessment of the emerging therapy to be as seamless as possible following the phase II Bloodwise TAP CLARITY Trial, the amendment was planned and funding applications submitted approximately 18 months prior to the availability of the CLARITY trial safety and activity data. At the time of starting the process, there was evidence of activity and safety of I+V in mantle cell lymphoma, but the combination had not yet been assessed in CLL. Due to the length of time it takes to obtain funding and approvals, the amendment was set in motion with the caveat that the applications would be withdrawn and the amendment dropped if the phase II data was not acceptable. Any changes to treatment schedule or safety monitoring that were required for CLARITY would have also been implemented into the FLAIR amendment. Had the emerging results been unacceptable and the amendment dropped, there would have been an amount of work done that had taken place unnecessarily, but this is similar to the risks associated with any new trial grant application. Work on protocol development and other amendment processes was started before contracts with the pharmaceutical funders were signed which also presented a financial risk; however, this was felt to be acceptable based on the preliminary approvals from both companies.

The decision to progress the FLAIR amendment was made 2 years before the amendment opened. This length of time was necessary for an amendment this substantial, although this may not be anticipated by collaborators. Many of the trial management processes are similar to those in setting up a new trial and take almost as long to implement, especially where funding needs to be sought.

### Operational challenges

Planning and setting-up such a substantial amendment at the same time as running the large, fast-recruiting phase III trial presents challenges. In terms of resource management, it is equivalent to running a large trial at the same time as setting up a separate trial which significantly impacts the workload for a single dedicated trials team. It was necessary to increase staffing and to have some staff moved off other projects to focus solely on FLAIR. As an indicator of resource required, the overall staff full-time equivalent (FTE) for the amendment was equivalent to the FTE in the original design, therefore doubling the number of staff needed to successfully deliver the trial. The drivers for this were the increase in sample size from 754 to 1516, the extended trial duration, increase in the number of participating sites and complexities of the new design such as additional IMP management. Defining the requirements of the original trial alongside the amendment and balancing between those priorities required active management and flexibility by the trial team. It was essential for the team to remain mindful of the existing trial timelines, and not inadvertently delay milestones due to the additional workload.

There was a new feasibility consultation performed with existing trial sites early on in the amendment process, rather than assuming enthusiasm. Larger centres and those recruiting well were generally very positive and reported finding it easier to implement than opening a new trial as they already had momentum and familiarity with the systems. In addition, having a higher chance of participants being allocated an experimental treatment was appealing. However, as the new drug was a higher risk, it meant a few smaller sites were not able to participate and subsequently withdrew from the trial. The FLAIR team thank all participating sites for their dedication and the time and effort they put into making this amendment a success.

### Planning for future potential changes in practice

One of the concerns with long trials, particularly those with different hypotheses being assessed at different times such as in platform designs, is that practice will change and the outcomes become less relevant or the standard control therapy will be superseded. In order to pre-empt and protect against this, a single-agent ibrutinib control arm was added concurrently to the I+V arm, so two different control groups were included. It is somewhat unusual for a confirmatory trial to include two control groups, and clearly, the number of patients needed is increased compared to a standard randomised controlled trial. However, there was reasonable evidence that the standard therapy could change over the life of the trial, so this measure was felt to be necessary. The strategy of having two controls allowed I+V to be assessed against the most relevant therapies without delaying the research. During the course of the trial, if FCR was superseded, it could be dropped from the randomisation without compromising the trial outcomes.

A key point raised within reviewers’ comments was that the trial is not designed to report confirmatory comparisons between the non-concurrent arms: I monotherapy and IR; or IR and I+V. Trials comparing I and IR were ongoing [21, 30], and whilst early evidence suggested that IR is not better than I, if further evidence emerges that shows otherwise, it is possible to directly compare endpoint data between the 122 contemporaneous patients for each comparison as an exploratory investigation to inform future trial designs. In addition, we would have data on MRD negativity, treatment duration and safety rates as well as health economic evaluations for single-agent I, IR and I+V from within the FLAIR trial to input into this assessment. The outcomes from non-contemporaneous trial arms will be treated as they would be had they arisen from different trials.

## Conclusions

We have described how the FLAIR trial was able to successfully provide a platform for an emerging new therapy to be assessed within an existing confirmatory trial framework. It is demonstrated that despite some challenges, this type of adaptation is feasible, acceptable and can give statistically unbiased outcomes. In addition, any logistical challenges were not insurmountable. This strategy offered substantial gains in efficiency for assessing the emerging therapy. For the original trial, it took over 2.5 years from submission of the outline funding application to the first centre opening, and 3.5 years for all centres to be open, which is not unusual for a confirmatory trial. By amending FLAIR rather than planning a new trial, the new hypotheses were incorporated almost seamlessly following on from the external phase II assessment, completely eliminating the time period between confirmatory trials. Due to opening additional centres even before the amendment was implemented, the original hypothesis is not delayed in recruitment or reporting. The primary assessment for the new hypothesis is planned just 1 year later than for the original, which is a saving of many years over planning and running a new trial. In addition, these hypotheses are able to be assessed in the same population at the same time without competing with one another.

The key benefit of this type of amendment relates to efficiency rather than necessarily being financial. Whilst there are some wider cost savings linked to reduced centre set-up times, some oversight functions and site monitoring, for example, the savings are hard to quantify and certainly would not be a driver in the decision to amend a trial in this way. Understanding the efficiencies of platform trial design warrants further investigation, and integration of data from other platform trials would be beneficial in this area.

The ability to amend FLAIR by adding new treatment arms has greatly benefitted patients because they have access to the latest therapies sooner. This was discussed in an article in the BBC News [31] which describes the extremely promising results from the I+V investigation in the phase II CLARITY trial, and how the use of a flexible trial design enabled this treatment to be quickly incorporated into a randomised confirmatory trial. The Trial Management Group continues to explore opportunities to further adapt the trial in response to emerging evidence in the field. A treatment arm for patients with genetically high-risk CLL is under development and would broaden the eligibility criteria of the trial to allow access for a patient group which are excluded from many clinical trials.

The FLAIR amendment has demonstrated that adapting a trial by adding experimental arms is feasible in practice without compromising the statistical validity or logistical integrity of the trial.

## Data Availability

LICTR will control the final trial dataset, and any requests for access will be reviewed by the TMG and TSC, subject to existing contractual arrangements with the funders. The protocol, sample case report forms and participant information are available on a case-by-case basis as agreed by the TMG, upon request to the corresponding author.

## Abbreviations

CID: Complex innovative design
CLL: Chronic lymphocytic leukaemia
CRFs: Case record forms
CSG: Clinical Studies Group
CRUK: Cancer Research UK
CTAAC: Clinical Trials Advisory and Award Committee
DMEC: Data Monitoring and Ethics Committee
FCR: Fludarabine, cyclophosphamide and rituximab
FTE: Full-time equivalent
FWER: Family-wise error rate
I: Ibrutinib
I+V: Ibrutinib and venetoclax
IMP: Investigational Medicinal Product
IR: Ibrutinib and rituximab
LICTR: Leeds Institute of Clinical Trials Research
MHRA: Medicines and Healthcare Products Regulatory Authority
MRD: Minimal residual disease
NCRI: National Cancer Research Institute
NRES: National Research Ethics Service
OS: Overall survival
PFS: Progression-free survival
PIS: Participant information sheet
PPI: Patient and public involvement
QoL: Quality of life
R: Rituximab
RCT: Randomised controlled trial
TAP: Trials Acceleration Programme
TMG: Trial Management Group
TSC: Trial Steering Committee
UK: United Kingdom
V: Venetoclax
